# Reappraisal of Leishmanin Skin Test (LST) in the management of American Cutaneous Leishmaniasis: A retrospective analysis from a reference center in Argentina

**DOI:** 10.1371/journal.pntd.0005980

**Published:** 2017-10-05

**Authors:** Alejandro Javier Krolewiecki, Maria Cristina Almazan, Marcelo Quipildor, Marisa Juarez, Jose Fernando Gil, Marco Espinosa, Maria Canabire, Silvana Pamela Cajal

**Affiliations:** 1 Instituto de Investigaciones en Enfermedades Tropicales, Universidad Nacional de Salta/CONICET, Oran, Argentina; 2 Division Infectologia, Hospital San Vicente de Paul, Oran, Argentina; 3 Division Pediatria, Hospital San Vicente de Paul, Oran, Argentina; US Food and Drug Administration, UNITED STATES

## Abstract

*Leishmania* (*Viannia*) *braziliensis* is the species most frequently implicated with cutaneous and mucosal leishmaniasis in the Americas; its diagnosis is based on the identification of amastigotes in lesions, which is limited by low parasite burden. Leishmanin Skin Test (LST) is a support tool for diagnosis, based on delayed type hypersensitivity responses to *Leishmania* antigens injected intradermally, used in endemic areas as a complement to diagnosis. A retrospective analysis of individuals evaluated for their first episode of tegumentary leishmaniasis at a reference center in Argentina during the period 2006–2015 was performed, with the goal of assessing its usefulness as a support tool in the diagnosis of leishmaniasis. Demographic, clinical and diagnostic work-up were analyzed in individuals with clinically compatible lesions, lesion`s smear and LST. A total of 733 cases that met the case definition were included in the analysis; 678 (93%) localized cutaneous cases, 50 (7%) with mucosal involvement and 5 (<1%) disseminated. Diagnostic confirmation was reached in 474 (65%) cases through positive smears from skin or mucosal lesions, with only 6 cases among this group having negative LST. Among smear negative cases, 190 were negative also by LST, but in 69 instances LST was positive. Across age groups, similar ratios of sensitivity between smear and LST were calculated. Lesions older than 21 days-old were found to correlate with positive results both for smear and LST significantly more than younger lesions. These findings support the clinical use of LST as a diagnostic complement for American Cutaneous Leishmaniasis across all age groups even in endemic areas. In this analysis, the correlation with smear was high. Standardization of this technique and further research into its most adequate preparation and utilization protocols across different sites will help in the management of suspicious clinical cases.

## Author summary

American Cutaneous Leishmaniasis (ACL) is a vector borne Neglected Tropical Disease with skin ulcers as the main clinical manifestation. Diagnosis is suspected when characteristic lesions occur in patients in areas known to be endemic and confirmed with microscopic identification of amastigotes in samples obtained from lesions. The Leishmanin Skin Test (LST) is an aid in the diagnosis based on the elicitation of delayed type hypersensitivity responses upon exposure to *Leishmania* antigens injected intradermally into the patient´s forearm and measured as the induration size after 48 hours. This analysis of 733 cases, which is the largest series of cases evaluating the role of LST in ACL highlights and establishes the usefulness of this complementary diagnostic tool with a strong correlation with lesion smear and a demonstration that false positive responses due to previous exposure to the parasite are not of concern. The results support the use of LST in endemic areas to assist in guiding clinical decisions regarding treatment initiation.

## Introduction

The leishmaniases are a group of vector borne neglected tropical diseases affecting a significant number of people in the tropical and subtropical regions of the world; this population is estimated in over 398 and 556 million people at risk of cutaneous leishmaniasis and visceral leishmaniasis respectively, in high burden countries [1]. As stated in a Technical Report gathered by WHO, there are dire needs for expansion of control programs and generation of research based evidence in all aspects of this group of diseases [2]. *Leishmania (Viannia) braziliensis*, which is distributed in the Americas, from Argentina to Mexico, has a significantly distinctive clinical behavior, being the most frequent cause of American Tegumentary Leishmaniasis (ATL), the clinical form that involves skin and/or mucosa in the Americas. Another feature of the leishmaniasis caused by *L*. *(V*.*) braziliensis* is the low burden of parasites in the lesions, which complicates the diagnosis through methods that depend on the timely identification of parasites from the lesions (direct methods) [3,4]; this scarcity is more pronounced in mucosal than in cutaneous lesions [5].

Northern Argentina has been endemic for tegumentary leishmaniasis in restricted areas since its initial descriptions in the 1910s, and remains endemic in those same areas, with evidence pointing towards urban transmission and the introduction of visceral disease due to *L*. *(Leishmania) infantum* mainly in the northeast but also in the northwest [6–8]. The Province of Salta, in the Northwest, has been the area of the country with the highest incidence of tegumentary leishmaniasis, with most cases originating in the Oran Department, where cutaneous and mucosal disease have been documented to be almost uniformly due to *L*. *(V*.*) braziliensis*; with rare cases due to *L*.*(V*.*) guyanensis* and *L*.*(L*.*) amazonensis* [9–11].

The diagnosis of tegumentary leishmaniasis relies mostly on the sum of epidemiologic background, clinically compatible lesions and confirmation through a direct test, which most frequently is a Giemsa stained smear from skin scrapping that is evaluated under immersion oil microscopy and identifies amastigotes with its characteristic kinetoplast. Alternative approaches include skin biopsies and needle aspirates for culture and most recently molecular biology approaches to detect *Leishmania* DNA from scrapings, aspirates and biopsies [12].

The Leishmanin Skin Test is a complementary tool for diagnosis that relies on the elicitation of delayed type hypersensitivity by the intradermal inoculation of *Leishmania* extracts, which provokes in the sensitized host, an immune response upon exposure to specific antigens and clinically manifests as an indurated nodule that can be seen and measured 48 hrs. after inoculation in the forearm. The LST was initially described by Montenegro and its principle is analogous to the Tuberculin test used for tuberculosis [13].

The purpose of this study was to perform a descriptive and comparative analysis of LST reactivity in suspected cases of ATL in an endemic area in a set of clinical and demographic variables.

## Methods

### Study location

The Instituto de Investigaciones en Enfermedades Tropicales (IIET) in the city of Oran (Oran Department), Northwestern Argentina, located at 23°08’S, 64°20’W, serves as a regional reference center for the diagnosis of leishmaniasis; patients are referred by local physicians or self-referred for the evaluation of lesions suspicious of leishmaniasis, which if epidemiologically and clinically compatible undergo diagnostic sampling through skin scrapping and LST. Upon completion of the diagnostic work-up, patients are referred to their physicians or the local reference Hospital.

An *ad-hoc* database was constructed with the information from the archives of the IIET, where in a de-identified manner, the following variables were included: age at presentation, sex, department of residence, lesion age, clinical form (cutaneous, mucosal involvement, disseminated), lesion/s location, largest lesion size, number of lesions, smear result, LST result. The case definition for this analysis was established as any individual with first episode of lesions compatible with ATL, which had a diagnostic work-up and results of smear and LST available for evaluation. The study was performed with cases seen at the IIET between January 1^st^, 2006 and December 31^st^, 2015. When smear results were >24hours older than the LST application, the case was excluded in order to prevent the bias induced by repeated smears in LST positive cases.

### Diagnostic procedures

Clinical diagnosis was made on suspicious cutaneous and mucosal lesions and categorized as “Localized cutaneous” form when all lesions were in the skin without meeting the case definition for “Disseminated” form, which was defined as ≥10 lesions in ≥2 body areas [14]. Mucosal disease was defined as compatible lesions in mucosal tissues (whether without cutaneous lesions “Mucosal” or concomitant with skin lesions “Mucocutaneous”).

As per the Standard Operating Procedures, the lesions were processed as follows: lesions with clinical and epidemiologic features of ATL were investigated through scrapping of the border of the lesion. The obtained sample was allowed to dry, fixed, stained with 10% Giemsa and examined under immersion oil optic microscopy for the presence of amastigotes. Semi-quantification of the burden of amastigotes was carried out as follows: negative, no amastigotes on the entire slide; +, 1–10 parasites/ 1000 fields; ++, 1–99 parasites/ 100 fields; +++, ≥10 parasites/ 10 fields. LST was applied by injecting intradermally 0.1 ml of Leishmanin (40μg of protein/ml) into the forearm, and the reaction recorded prior to the initiation of treatment at 48 to 72 hours post application; indurations ≥5mm were considered positive [15].

### LST preparation

LST was locally prepared with a soluble extract of promastigotes of *L*.*(V*.*) braziliensis*, obtained in culture from a patient of our region (strain MHOM/AR/03/OLO1). Parasites were identified through Multi *Locus* Enzyme Electrophoresis. During study period, two different lots of LST were employed; these, were produced from the same strain and following the same protocol.

The cultures were initially maintained in biphasic agar-blood supplemented with 20% defibrinated rabbit blood (UNSa animal facilities, Salta, Argentina) and LIT medium supplemented with 10% Fetal Bovine Serum (Sigma-Aldrich). Promastigotes were harvested at peak log phased growth, centrifuged at 2500 rpm for 10 minutes at 4°C and washed with saline solution. Afterwards, promastigotes were re-suspended at a concentration of 10^6^ /mL in a phenol 5‰ solution; this solution was centrifuged at 3000 rpm for 15 minutes at 4°C. The obtained supernatant was sterilized with filters of 0.22μm pore (Merck Millipore, Billerica MA) and stored at -20°C until use.

### Data analysis

With the database filled, conflicting entries clarified and quality control measurements approved, the data was locked and analyzed through Epidat (Xunta de Galicia, Spain). Dichotomous variables were analyzed through Chi square (X^2^) and the Yates`correction for continuity was added for calculations in groups with >40 cases; gamma and Taub-C tests were used for variables organized in ordinal categories; agreement between tests was also measured with the Kappa Index. Continuous variables were analyzed according to the underlying distribution of the data using Student`s T test or the Mann-Whitney´s non-parametric test. Correlations were estimated from Spearman´s rank correlation coefficients. Significance was defined at p values ≤0.05.

### Ethical considerations

All the database was de-identified. The project was evaluated and approved by the Bioethics Committee of the Universidad Nacional de Salta as part of the research plan upon entry to CONICET Research Track of the first author (AJK).

## Results

The analysis included 733 cases that met the entry criteria. From 730 cases with information on the locality of residence, 665 (91.1%) were form Oran Department and another 43 (5.9%) from other Departments within Salta Province and 22 (3%) from other parts of Argentina or Bolivia. The male:female ratio was 3:1. All cases had information on age, with a median (inter quartile range) of 42 years old (IQR: 28–57) and no differences between females and males; only 24 cases (3.3%) corresponded to individuals ≤14 year-old. Description of clinical presentation is detailed in Table 1.

**Table 1:** Clinical description of the study population.

Clinical presentation (733 cases with data)	Localized cutaneous: 678 (92.5%)
	Mucosal involvement: 50 (6.8%)*
	37 (5%) muco-cutaneous
	13 (1.8%) mucosal
	Disseminated: 5 (0.7%)
Number of cutaneous lesions (684 cases with data)	Single: 528 (77.2%)
	2: 91 (13.3%)
	3: 32 (4.7%)
	4: 10 (1.5%)
	≥5: 23 (3.3%)
Localization of cutaneous lesions (697 localizations among 693 cases with data)	Head & neck: 97 (13.9%)
	Upper extremities: 203 (29.1%)
	Lower extremities: 333 (47.8%)
	Trunck: 52 (7.5%)
	Pelvis: 12 (1.7%)

*: all mucosal lesion involved nasal mucosa

The diagnostic procedures showed that 474 of the 733 cases with clinically suspicious lesions (64.7%) had diagnostic confirmation through positive smears. When discriminated by the clinical form of presentation, the 474 positive smears came from 436 localized cutaneous, 34 mucosal and 4 disseminated cases. In this analysis, considering the whole study population, there was agreement between tests in 658 of 733 cases (89.8%) which includes 468 cases with both positive tests and 190 with negative results on LST and smear; disagreement in the remaining 75 cases included only 6 (0.8%) cases corresponding to positive smears with negative LST (5 cutaneous and 1 mucosal) and 69 cases with positive LST and negative smear; the Kappa index agreement between smear and LST was 0.76 (p <0.001) (Table 2). When analyzed in subgroups by clinical presentation, the agreement remained significant in all groups through the application of Kappa index and X^2^ with Yates correction, including the subgroup of disseminated cases with only 5 cases and a full agreement between tests (Table 2). Sex and age of the lesion were found not to be associated with clinical form; there was however a significantly higher proportion of positive cases among males both for LST and smear results. The prevalence of positive LST and smear results among the study population was stable across age groups, as was the relationship between LST and smear positivity (Table 3).

**Table 2:** Contingency tables comparing agreement between smear and LST among different clinical forms of American Cutaneous Leishmaniasis.

Clinical presentation			LST		Total
			Neg	Pos	Neg
Cutaneous*	Smear	Neg	183	59	242
		Pos	5	431	436
	Total		188	490	678
Mucosal involvement**	Smear	Neg	6	10	16
		Pos	1	33	34
	Total		7	43	50
Disseminated***	Smear	Neg	1	0	1
		Pos	0	4	4
	Total		1	4	5

*: Kappa Index: 0.78 (p<0.001). X2 and Yates`correction: p<0.001.

**: Kappa Index: 0.41 (p = 0.01). X2 p = 0.001, and Yates`correction: p = 0.004

***: Kappa Index: 1 (p = 0.025). X2 p = 0.025, and Yates`correction: p = 0.4

**Table 3:** LST and smear results in the different age groups. LST_prev_: prevalence of LST positive cases. SMEAR_prev_: prevalence of lesion’s smear positive cases.

Age group (years)	n	LST		Smear		LST_prev_/SMEAR_prev_
		Neg	Pos	Neg	Pos	
0–10	6	1	5	1	5	83%/83%: 1.00
11–20	71	16	55	22	49	77%/69%: 1.12
21–30	151	37	114	49	102	75%/68%: 1.12
31–40	125	26	99	34	91	79%/73%: 1.09
41–50	106	23	83	28	78	78%/74%: 1.06
51–60	132	43	89	52	80	67%/61%: 1.11
>60	142	50	92	73	69	65%/49%: 1.33
Total	733	196	537	259	474	73%/65%: 1.13

**Fig 1:**
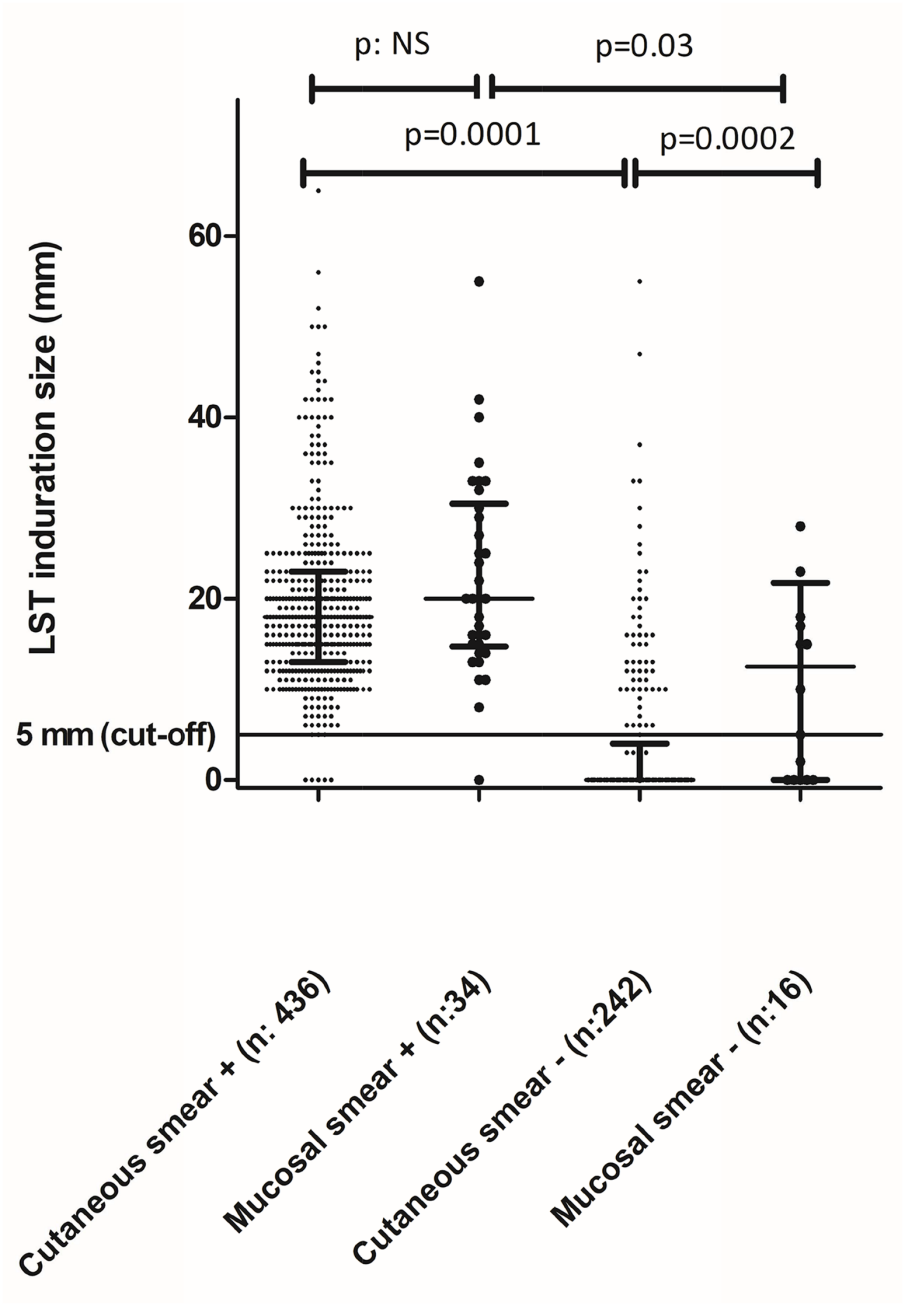
Leishmanin Skin Test (LST) induration among 733 individuals with diagnostic work up classified according to clinical form and result of lesion’s smear. Median and inter quartile range error bars drawn for each group. P values between groups calculated with Mann Whitney´s test.

The LST response measured through the size of induration had a median (IQR) of 18mm (13–23) among smear positive cases and 0mm (0–0) among those with a negative smear. Comparing median values of induration size according to clinical form and smear results demonstrated significant differences between smear categories but also between cutaneous and mucosal lesions among smear negative groups (Fig 1). Correlations between parameters of disease severity and LST size of induration were evaluated through the analysis of correlation between induration size and the diameter of the largest lesion and with the semi-quantitation of the parasite burden in the smear; the size of the largest skin lesion, which was available for analysis in 657 cases, showed a significant positive correlation in the Spearman correlation test (p = 0.001). We also found a significant positive correlation between semi-quantitative parasite burden in the lesion and LST positivity (p<0.001), with all cases showing +++ lesions (75 cutaneous, 1 mucosal and 3 disseminated) having a positive LST. Besides the cut-off of 5mm of induration defining positivity of LST, the absolute induration size was also found to be significantly correlated with parasite burden (p<0.001).

The age of the lesion was analyzed in view of the influence that the time to mount an adequate immunologic response might pose on the performance of the LST. With a median time of lesion age of 30 days among patients with cutaneous lesions, a cut-off of 21 days was used to compare lesions defined as “recent” vs “old”. In this analysis, we found that among the 45 cases with mucosal involvement and information of lesion age, only 1 corresponded to “recent lesion”, as expected; therefore, we concentrated this analysis on localized cutaneous cases where we identified a total of 674 cases, 236 (35%) were recent and the other 438 (65%) late. Lesions older than 21 days had a significantly higher likelihood of having a positive LST (Table 4). When 30 days was used to separate the 2 groups of lesions, the difference was no longer statistically significant. Potential confounding variables like differences in time to seek for medical attention between males and females were ruled out, although there was a significant correlation between age of the patient and lesion age in Spearman’s correlation test (p = 0.02) among these 674 localized cutaneous cases. We also identified a statistically significant increase in the positive results in lesion`s smear when performed in the group of lesions older than 21 days (p = 0.001) among the same subgroup of cases. The localization of the lesion was evaluated for cases with localized cutaneous disease as a variable in reference to the results of LST and smear; among 341 cases with localized cutaneous lesions below the waist, Spearman`s correlation between LST and smear remained significant (p<0.001). In the subgroup of 117 cases of localized cutaneous lesions below the waist and <21 days of lesion age, 52 cases (44%) had confirmed amastigotes on smear of the lesion, of which 51 also were LST+, and another 10 cases (8.5%) were LST+ with negative smears.

**Table 4:** Variations in Leishmanin Skin Test (LST) reactivity and lesion’s smear result according to lesion age of > or ≤ 21 days among 674 individuals with cutaneous lesions without mucosal involvement. P values between groups calculated with Chi square test(X^2^) and the Yates`correction for continuity.

		≤21-day-old lesion	>21-day-old lesion	Total
LST (*)	+	151 (31%)	337 (69%)	488
	-	85(45.7%)	101 (54.3%)	186
Smear (**)	+	132 (30.3%)	303 (69.7%)	435
	-	104 (43.5%)	135 (56.5%)	239

*: p<0.01

**: p = 0.01

## Discussion

The findings of this study highlight the usefulness and contribution of LST in the diagnosis of ATL in a highly endemic area. Currently, there is a lack of standardized procedures for LST preparation; studies are necessary to harmonize key aspects of this diagnostic method if a wider use for the management of suspected clinical cases is expected. Recently published guidelines on the management of leishmaniasis discourage the use of LST due to its unavailability in the US and Canada [12], a situation which coupled with its continuous use in countries endemic for ATL in Latin America, should encourage further research, standardization and production in order to make it widely available for clinical use and epidemiologic investigations.

This study, with a large sample size of over 700 patients, contributes to a better understanding of the characteristics of this type of diagnostic tool. This becomes relevant and important in low-resource settings where molecular amplification assays are not routinely available. These results also contribute as a complement in diagnosis even in well-equipped centers where PCR and microscopy might not be able to confirm the diagnosis.

To our knowledge, this is the largest study analyzing retrospectively the role of LST in clinical practice. The results are most significant at confirming the high sensitivity and specificity of this method even in a highly endemic area as ours, where although the exposure risk is significant for most of the population due to the rural and urban transmission of leishmaniasis [6], the correlation between lesion smears and LST was highly significant. This was seen for all the clinical forms of the disease, including localized cutaneous, mucocutaneous and disseminated leishmaniasis; showing an overall sensitivity of over 98% using lesion’s smears as the standard comparator. The maintenance of the ratio of positive LST to positive smears across age groups (Table 3), suggests that the group of cases with positive LST and negative smears represents a clinically significant contribution for the diagnosis of leishmaniasis, rather than LST sensitization due to exposure to the parasite but without clinical signs of infection. With 13% of the total LST positive cases having negative smears, although this most likely represents the limits of sensitivity of microscopy when done by expert observers [5,16], alternative diagnoses must be considered an included in the diagnostic work-up; some of them, like sporotrichosis, with unclear cross reactivity in LST responses [17]. Previous studies that used the LST as a marker of exposure/infection in the general population, have noted that the prevalence of LST+ cases increases with age at a disproportionally higher rate than the increases in the incidence of clinical disease [8], referring to the cumulative risk of exposure as a function of time, rather than just the poor sensitivity of the direct methods. The sensitivity of the LST found in this study is in accordance with previous studies, all of them informing sensitivities ≥90% [18,19]. The size of the induration was not found to be larger among cases with mucosal involvement in accordance with previous smaller studies [20] although other small studies found larger induration size in cases with mucosal involvement [21,22].

Previous studies have looked at the impact of lesion age in reference to the sensitivity of LST with results suggesting that LST was less sensitive in lesions of <1 month [20,23]; our study allowed us to determine that lesions of <21days old were significantly less likely to produce a positive LST (Table 4). This finding has significant clinical implications in order to define the best timing for the use of the LST. Still, among cases with lesions <21 days old, the most frequent diagnosis based on positive smears was leishmaniasis with 132 (56%) of 236 cases (Table 4). We also identified statistically significant differences in lesion smears, with lesions >21 days being more likely to render positive results; previous studies have noted decreasing sensitivity of the lesion smears in lesions >3 months old [5,23]. The most likely explanation for the presence of more cases with negative LST and smears in those lesions <21 days old and below the waist is that in those cases alternative etiologies that often resolve within 3 weeks are more frequent. The results on the analysis based on the localization of the lesions revealed that the presence of lesions above or below the waist does not alter the correlation between LST and smear. All this information supports the clinical conduct of indicating anti-Leishmania therapy when typical lesions and a positive LST are present despite the lack of parasitologic confirmation of leishmaniasis, in the context of an adequate epidemiologic background and a diagnostic evaluation that rules out other pathologies like vascular and traumatic ulcerative lesions, foreign-body reactions, superinfected insect bites, myiasis, impetigo, fungal and mycobacterial infections, sarcoidosis, and neoplasms for cutaneous lesions and paracoccidiodomycosis, histoplasmosis, syphilis, tertiary yaws, leprosy, rhinoscleroma, midline granuloma, sarcoidosis, and neoplasms for mucosal lesions [24].

The limitations of this study include its retrospective nature, the lack of a diagnostic gold standard, the lack of validated protocols that formally evaluate stability and reproducibility of the LST preparation, and the fact that the homology between the circulating strains in our area and the strain used to produce LST are likely to be optimal, although at least 2 genotypes of *L*. *(V*.*) braziliensis* have been documented to circulate in the study area [25]; therefore, the reproducibility of this results in a less than optimal situation need confirmation based on differences seen in previous studies [18]; despite this, epidemiologic studies performed in our region with LST produced in other regions were still useful and current developments are still unclear on the impact of the genetic difference between *Leishmania* parasites on various aspects of immunology and pathogenesis [8,26].

In summary, LST is an adequate and useful diagnostic complement for the diagnosis of ACL. Further work on its standardization and validation across geographic locations and including correlation with molecular biology methods in the diagnostic panel is warranted in view of its performance and ease of use in clinical settings.

## Supporting information

S1 Dataset (XLSX)

S2 Dataset Coding. (DOCX)
