# Supplementary material for: Reappraisal of Leishmanin Skin Test (LST) in the management of American Cutaneous Leishmaniasis: A retrospective analysis from a reference center in Argentina
Source: PLoS Negl Trop Dis. 2017 Oct 5;11(10):e0005980. doi: 10.1371/journal.pntd.0005980 (PMC5645152; doi:10.1371/journal.pntd.0005980)
Supplement: S2 Dataset — (DOCX) [file pntd.0005980.s002.docx]

DATASET CODIFICATION:

Sex: 1=male; 2=female

Procedence: 1=Oran department; 2=other department in Salta Province; 3=Other provinces in Argentina; 4=Bolivia

Clinical form: 1=cutaneous; 2=pure mucosal; 3=muco-cutaneous; 4=disseminated

Lesion site: 1=head & neck; 2=upper extremities; 3=lower extremities; 4=trunck; 5=pelvis

Smear result: 0=negative; 1=+; 2=++; 3=+++
